# Excretions/Secretions from Bacteria-Pretreated Maggot Are More Effective against *Pseudomonas aeruginosa* Biofilms

**DOI:** 10.1371/journal.pone.0049815

**Published:** 2012-11-30

**Authors:** Ke-chun Jiang, Xin-juan Sun, Wei Wang, Lan Liu, Ying Cai, Yin-chen Chen, Ning Luo, Jian-hua Yu, Da-yong Cai, Ai-ping Wang

**Affiliations:** Department of Endocrinology, 454 Hospital of PLA, Nanjing, Jiangsu Province, China; University of Iowa Carver College of Medicine, United States of America

## Abstract

**Background:**

Sterile larvae—maggots of the green bottle blowfly *Lucilia sericata* are employed as a treatment tool for various types of chronic wounds. Previous studies reported that excretions/secretions (ES) of the sterile larvae could prevent and remove the biofilms of various species of bacteria. In the present study we assessed the effect of ES from the larvae pretreated with *Pseudomonas aeruginosa* on the bacteria biofilms.

**Methods and Findings:**

We investigated the effects of ES from the maggot pretreated with *P. aeruginosa* on the biofilms using microtitre plate assays and on bactericidal effect using the colony-forming unit (CFU) assay. The results showed that only 30 µg of the ES from the pretreated maggots could prevent and degrade the biofilm of *P. aeruginosa*. However, the CFU count of *P. aeruginosa* was not decrease when compared to the ES from non pretreated maggots in this study condition. It is suggested that the ES from the pretreated maggot was more effective against biofilm of *P. aeruginosa* than sterile maggot ES.

**Conclusions:**

Our results showed that the maggot ES, especially the bacteria-pretreated larva ES may provide a new insight into the treatment tool of the bacterial biofilms.

## Introduction

As the population ages, the number of patients suffering from chronic wounds attributable to diseases such as diabetes mellitus and peripheral vascular disease is on the rise [1]. The healing process is often complicated by bacterial infections on the wound surface [2], especially when the bacteria are residing in biofilms [3]. Biofilm bacteria exhibit altered growth characteristics and gene expression profiles as compared with those planktonic in the environment [4].An important consequence following biofilm formation is that the bacteria are protected against the actions of antibiotics and the effecter molecules of the immune system [5], [6].

Sterile larvae of the green bottle blowfly *Lucilia sericata* are used as a treatment tool for various types of chronic wounds [7]. In clinical practice, fast healing of infected wounds by means of maggot debridement therapy (MDT) in combination with antibiotics has been observed [8]. The molecules involved in these actions are believed to be contained in the excretions/secretions (ES) of maggots. Recently, researches showed that Sterile Maggot ES could effectively perform against biofilms of *S. aureus* and *P. aeruginosa* [9], [10], [11]. However, in MDT, once the sterile larvae are applied to an infected wound, they are no longer germ-free, becoming infected state without physical injury. Previous studies have described that the antibacterial capacities of the infected larvae were better than those of sterile larvae [12]. Since modulation of bacterial biofilms will have a major impact on the healing process of infected wounds, we assessed the effect of ES from *P. aeruginosa-* infected maggot (pretreated ES) on the formation of biofilms and on the disruption of established biofilms of the bacteria.

## Materials and Methods

### Maggots and maggot ES

ES of sterile second-and or third-instar larvae of *L.sericata* from our own laboratory was collected as described by van der Plas et al [13]. In short, 500 larvae were incubated in physiological saline for 60 min at 37°C, in darkness.

ES of pretreated larvae was collected as described by Basset et al [14]. Sterile third-instar larvae were incubated with different amounts of bacteria for 2 hours and then the larval ES was collected and centrifuged at 13,000× g for 10 minutes at 4°C to remove particulate material. Then, the supernatant was filtered with 0.22 µm filtration membrane and stored at −20°C or for use. The concentration of ES protein was determined using the Pierce BCA Protein Assay Kit (Pierce,USA) according to manufacturer's instructions.

### Bacterial strains and growth conditions

The strain of *P.aeruginosa* was isolated from patients of our department from infected wounds, and then grown in 3% Tryptone Soya Broth(TSB) at 37°C under vigorous shaking.

### Biofilm assay

Biofilm formation of *P.aeruginosa* in 96-well polyvinyl chloride plates was conducted as described by van der Plas et al [9]. In short, Bacteria from over night cultures were diluted with medium in 1∶100 and 5 µL aliquots of these bacterial suspensions were added to each well of the 96-well flat-bottomed microtiter plate, which contains 130 µL of the medium with or without ES. After 24 h incubation, planktonic cells were removed and the wells were washed with tap water. Subsequently, biofilms were exposed to a 1% crystal violet solution for 15 min, washed and then incubated in absolute ethanol for 15 min to extract the crystal violet retained by the cells. Next, this solution was measured at a wavelength of 590 nm to quantify the formed biofilm.

### Measurement of bactericidal effect *in vitro*

To determine the bactericidal effect of ES on planktonic bacteria, *P.aeruginosa* was incubated at 37°C with different concentrations of ES, or with sterile physiological saline as control. The aliquots of the samples were diluted with PBS after incubation for 24 h. Subsequently, 2 µL of the diluted solution were spread onto tryptone soya agar (TSA). After overnight incubation at 37°C, the number of colonies was counted manually.

### Microscopic analysis

Scanning electron microscope and bright field microscope were used for visualizing the biofilms in the absence or presence of pretreated larval ES in the culture medium.

### Statistical analysis

All statistical analyses were operated by GraphPad Prism software. One-way ANOVA and two-tailed Student t-tests were used in our statistical analysis, and SNK method was used for multiple comparisons. A P-value<0.05 was considered as statistically significant.

## Results

### Effect of pretreated ES on biofilms formation

**Figure 1:**
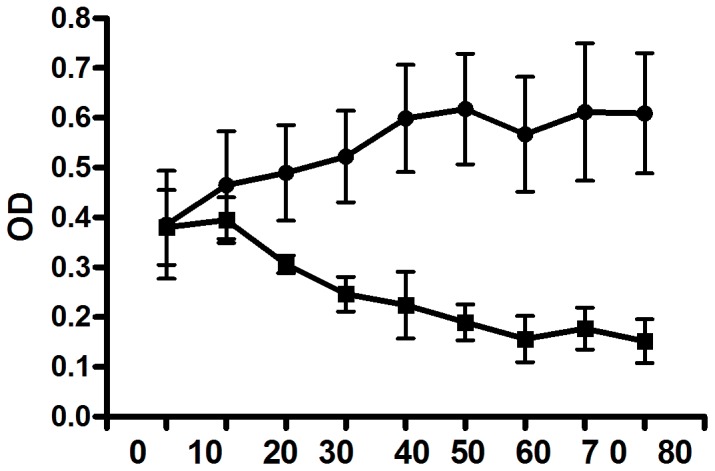
Effect of pretreated ES on biofilms formation. Bacterial suspensions were added to each well with pretreated ES or sterile ES. After 24 h incubation, biofilms were exposed to a 1% crystal violet solution, and the amount of biofilm was measured by its A590. For 20 µg on, all values are significantly (*p<0.05*) different from these for biofilms without pretreated(**-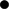-** nonpretreated group, **-**▪**-** pretreated with 10^6^ cfu/ml).

To find out whether pretreated ES can prevent biofilm formation, the bacteria-infected larva ES and bacterial suspensions were added to each well and then incubated for 24 h. The amount of biofilm was quantified by measuring the optical density. The result revealed that the amount of *P. aeruginosa* biofilm was CFU number dependently reduced (data not shown) and that in the 1×10^6^ CFU bacteria-pretreated group the biofilm amount was significantly lower than in the sterile group (Figure 1).

### Effect of pretreated ES on established biofilm

**Figure 2:**
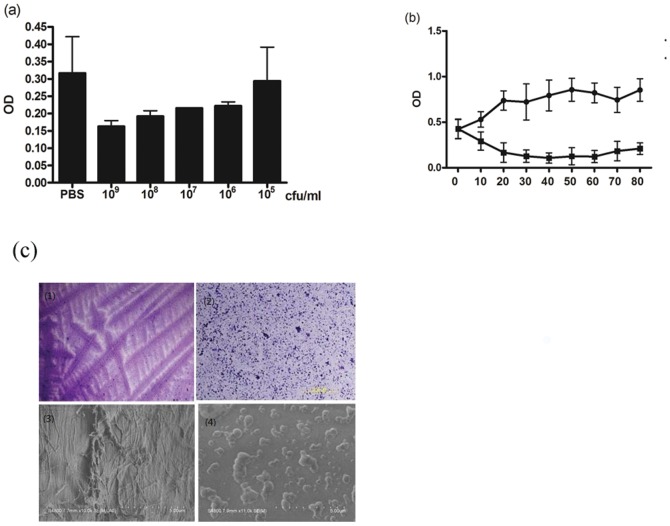
Effect of pretreated ES on established *P.aeruginosa* **biofilm.** (a) ES from different CFU of *P. aeruginosa* fed sterile larva, then added to the well which contained *P.aeruginosa* biofilm, 24 h incubation, biofilms were exposed to a 1% crystal violet solution, and the amount of biofilm was measured by its A590. (b) Different amounts of ES from 1×10^6^ CFU of *P. aeruginosa* fed sterile larva were added to the well which contained *P.aeruginosa* biofilm, 24 h incubation, biofilms were exposed to a 1% crystal violet solution, and the amount of biofilm was measured by its A590. For 30 µg on, all values are significantly (*p<0.05*) different from these for biofilms without pretreated.(**-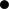-** nonpretreated group, **-**▪**-** pretreated with 10^6^ cfu/ml) (c) Light microscopic and scanning electron microscopic graphs of *P.aeruginosa* biofilm with or without infected ES. (c-1) Light microscopic graph of *P.aeruginosa* biofilm; (c-2) Light microscopic graph of *P.aeruginosa* biofilm which treated with infected ES ; (c-3) Scanning electron microscopic graphs of *P.aeruginosa* biofilm ;(c-4) Scanning electron microscopic graphs of *P.aeruginosa* biofilm which treated with infected ES.

To investigate the effect of pretreated ES on established biofilms, we fed the sterile maggot with different amounts of bacteria. The larval extracts were collected after the set periods of incubation with bacteria or PBS, and then the ES was added to the well with *P. aeruginosa* bioflilm. The result showed that the amount of *P. aeruginosa* biofilm in the 1×10^6^ CFU pretreated group was significantly lower than that in the PBS group (Figure 2a). Furthermore, after adding 30–80 µg of 1×10^6^ CFU/ml pretreated ES, the *P. aeruginosa* biofilm breakdown was dose-dependently enhanced (Figure 2b).

The effect of pretreated ES was also examined on *P. aeruginosa* bioflilm by light microscope and scanning electron microscope. It is demonstrated that the result was similar to that showing in Figure 1a. Both means after the pretreated ES treatment, the structure of *P. aeruginosa* biofilm was disrupted (Figure 2c).

### Effect of pretreated ES on *P. aeruginosa* growth

**Figure 3:**
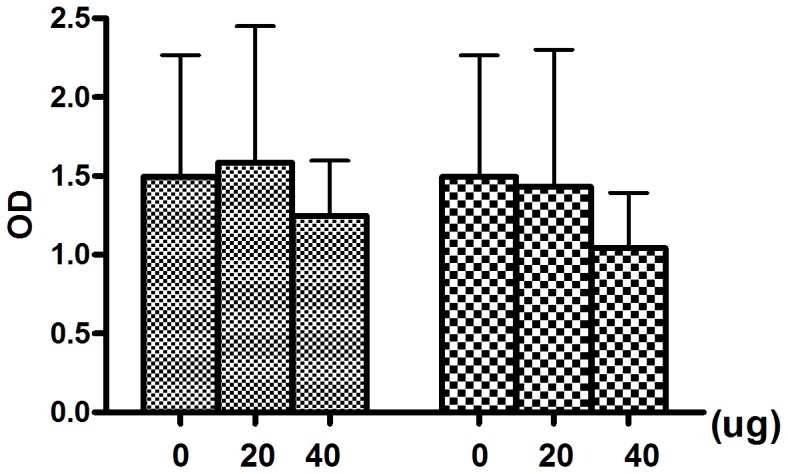
Antimicrobial activity of maggot ES against P. aeruginosa. The sterile larval were pretreated with 10^6^ cfu/mL *P. aeruginosa* or not, then 20 µg and 40 µg infected ES or sterile ES were added to each bacteria well respectively, 24 h later, the number of colony-forming unit was assayed(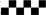 nonpretreated group, 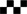 pretreated with 10^6^ cfu/ml).

It is reported that sterile larva ES may have bactericidal activities against Gram-positive and Gram-negative bacteria [15], [16], we determined the effect of the pretreated ES on the number of viable biofilm-associated *P. aeruginosa* in our experiments. The result demonstrated that using the current doses and conditions maggot ES did not reduce the total number of bacteria in the wells (Figure 3).

## Discussion

Bacteria within chronic wounds often reside in biofilms, which protect bacteria against the actions of antibiotics [4]. Previous reports showed that sterile maggot ES could be effective against biofilms of *P. aeruginosa* [9]. However, in clinical treatment, when sterile larvae are put of wound surface during MDT, they contact pathogenic bacteria and become non-sterile. Furthermore, the maggots of *Lucilia sericata* are successfully used as a treatment for infected wounds [17]. It is suggested that infected environment might not influence the efficiency of maggot ES against biofilms.

In this study, we demonstrated that ES from bacteria-pretreated larvae could also prevent and break down biofilm on *P. aeruginosa*. This conclusion is based on the following observations. First, only 30 µg pretreated maggot ES could break down established biofilms. Although a previous study showed that sterile maggot ES was effective against biofilms of *P. aeruginosa* [9], the pretreated maggot ES was considered to better reflect the context of an actual clinical wound [7]. Our results demonstrated that bacterial pretreatment of sterile larvae resulted in a dose-dependent increase in disrupting established biofilms. Secondly, the effect of 20 µg pretreated maggot ES, which could prevent biofilm formation is similar to that of sterile maggot ES [9]. Thus, the infection model is very similar to the clinical wound context in MDT and will be a powerful tool to study the activities of *L. sericata* larvae in MDT.

However, the antibacterial activity against *P. aeruginosa* was not detected in pretreated larva ES, which was pretreated with 1×10^6^ bacteria. Previously published studies indicated that the antibacterial activities induced by *S. aureus* and *P. aeruginosa* were effective against *S. aureus*, but not against *P. aeruginosa* [12], [15], [18]. Moreover, previous clinical studies showed that MDT was more effective in Gram-positive infected wounds than in Gram-negative infected ones [19], [20]. It is clear that maggot continuously secrete its product in wounds, but in our experiment we only added ES once to bacteria suspension. Therefore, It is presumed that more *P. aeruginosa* suspension would be needed to activate larval immune systems in future study.

## Conclusions

This investigation demonstrated that the bacteria-pretreated ES has a capacity to inhibit bioflim formation and break down existing biofilm more effectively. Although further studies are needed, these results suggest that bacteria-infected larva may induce new products to survive in such a harmful environment which is very similar to the clinical context in MDT. Further investigation would be needed to identify the bioactive compounds of infected larva ES, which may lead to better understanding the mechanisms of MDT [21].

## References

[1] Chan DC, Fong DH, Leung JY, Patil NG, Leung GK (2007) Maggot debridement therapy in chronic wound care. Hong Kong Med J 13: 382–386. 17914145

[2] Gjodsbol K, Christensen JJ, Karlsmark T, Jorgensen B, Klein BM, et al (2006) Multiple bacterial species reside in chronic wounds: a longitudinal study. Int Wound J 3: 225–231. 16984578 10.1111/j.1742-481X.2006.00159.x PMC7951738

[3] Edwards R, Harding KG (2004) Bacteria and wound healing. Curr Opin Infect Dis 17: 91–96. 15021046 10.1097/00001432-200404000-00004

[4] Stoodley P, Sauer K, Davies DG, Costerton JW (2002) Biofilms as complex differentiated communities. Annu Rev Microbiol 56: 187–209. 12142477 10.1146/annurev.micro.56.012302.160705

[5] Sheldon AT Jr (2005) Antibiotic resistance: a survival strategy. Clin Lab Sci 18: 170–180. 16134477

[6] Davis SC, Martinez L, Kirsner R (2006) The diabetic foot: the importance of biofilms and wound bed preparation. Curr Diab Rep 6: 439–445. 17118226 10.1007/s11892-006-0076-x

[7] Huberman L, Gollop N, Mumcuoglu KY, Block C, Galun R (2007) Antibacterial properties of whole body extracts and haemolymph of Lucilia sericata maggots. J Wound Care 16: 123–127. 17385589 10.12968/jowc.2007.16.3.27011

[8] Cazander G, Pawiroredjo JS, Vandenbroucke-Grauls CM, Schreurs MW, Jukema GN (2010) Synergism between maggot excretions and antibiotics. Wound Repair Regen 18: 637–642. 20946137 10.1111/j.1524-475X.2010.00625.x

[9] van der Plas MJ, Jukema GN, Wai SW, Dogterom-Ballering HC, Lagendijk EL, et al (2008) Maggot excretions/secretions are differentially effective against biofilms of Staphylococcus aureus and Pseudomonas aeruginosa. J Antimicrob Chemother 61: 117–122. 17965032 10.1093/jac/dkm407

[10] van der Plas MJ, Dambrot C, Dogterom-Ballering HC, Kruithof S, van Dissel JT, et al (2010) Combinations of maggot excretions/secretions and antibiotics are effective against Staphylococcus aureus biofilms and the bacteria derived therefrom. J Antimicrob Chemother 65: 917–923. 20189943 10.1093/jac/dkq042

[11] Harris LG, Bexfield A, Nigam Y, Rohde H, Ratcliffe NA, et al (2009) Disruption of Staphylococcus epidermidis biofilms by medicinal maggot Lucilia sericata excretions/secretions. Int J Artif Organs 32: 555–564. 19856274 10.1177/039139880903200904

[12] Kawabata T, Mitsui H, Yokota K, Ishino K, Oguma K, et al (2010) Induction of antibacterial activity in larvae of the blowfly Lucilia sericata by an infected environment. Med Vet Entomol 24: 375–381. 20946439 10.1111/j.1365-2915.2010.00902.x

[13] van der Plas MJ, van der Does AM, Baldry M, Dogterom-Ballering HC, van Gulpen C, et al (2007) Maggot excretions/secretions inhibit multiple neutrophil pro-inflammatory responses. Microbes Infect 9: 507–514. 17350304 10.1016/j.micinf.2007.01.008

[14] Basset A, Khush RS, Braun A, Gardan L, Boccard F, et al (2000) The phytopathogenic bacteria Erwinia carotovora infects Drosophila and activates an immune response. Proc Natl Acad Sci U S A 97: 3376–3381. 10725405 10.1073/pnas.070357597 PMC16247

[15] Kerridge A, Lappin-Scott H, Stevens JR (2005) Antibacterial properties of larval secretions of the blowfly, Lucilia sericata. Med Vet Entomol 19: 333–337. 16134984 10.1111/j.1365-2915.2005.00577.x

[16] Bexfield A, Nigam Y, Thomas S, Ratcliffe NA (2004) Detection and partial characterisation of two antibacterial factors from the excretions/secretions of the medicinal maggot Lucilia sericata and their activity against methicillin-resistant Staphylococcus aureus (MRSA). Microbes Infect 6: 1297–1304. 15555536 10.1016/j.micinf.2004.08.011

[17] Cazander G, van Veen KE, Bernards AT, Jukema GN (2009) Do maggots have an influence on bacterial growth? A study on the susceptibility of strains of six different bacterial species to maggots of Lucilia sericata and their excretions/secretions. J Tissue Viability 18: 80–87. 19362001 10.1016/j.jtv.2009.02.005

[18] Thomas S, Jones M, Shutler S, Jones S (1996) Using larvae in modern wound management. J Wound Care 5: 60–69. 8697135 10.12968/jowc.1996.5.2.60

[19] Steenvoorde P, Jukema GN (2004) The antimicrobial activity of maggots: in-vivo results. J Tissue Viability 14: 97–101. 15709356 10.1016/s0965-206x(04)43005-8

[20] Bowling FL, Salgami EV, Boulton AJ (2007) Larval therapy: a novel treatment in eliminating methicillin-resistant Staphylococcus aureus from diabetic foot ulcers. Diabetes Care 30: 370–371. 17259512 10.2337/dc06-2348

[21] Hoffmann JA (2003) The immune response of Drosophila. Nature 426: 33–38. 14603309 10.1038/nature02021

